# When a Child Refuses to Play: A Rare Myopathy

**DOI:** 10.7759/cureus.68372

**Published:** 2024-09-01

**Authors:** Luzia Condessa, Susana Dias, Sofia Moura Antunes, Mafalda Martins, Inês Madureira

**Affiliations:** 1 Pediatrics, Hospital de Cascais Dr. José de Almeida, Lisbon, PRT; 2 Pediatric Rheumatology, Hospital Dona Estefânia, Centro Hospitalar Universitário Lisboa Central, Lisboa, PRT

**Keywords:** dermatomyositis, polymyositis, idiopathic inflammatory myopathies, myositis, muscle weakness

## Abstract

Idiopathic inflammatory myopathies (IIM) are a rare group of systemic diseases characterized by progressive proximal muscle weakness and skeletal muscle inflammation. We describe a clinical report of a seven-year-old boy presenting with myalgia and proximal muscle weakness beginning three weeks earlier, with laboratory, MRI, and muscle biopsy findings consistent with IIM. The patient was treated with corticosteroids, methotrexate, immunoglobulin, and intensive motor rehabilitation, with favorable evolution. Diagnosis of Juvenile Polymyositis was confirmed. Three years later, we assisted a relapse of muscle weakness and muscle cytolysis with the onset of bilateral eyelid skin microulcers compatible with dermatomyositis. This report intends to highlight the importance of early diagnosis and treatment in IIM due to the significant burden associated with this group of diseases. In this case, the late onset of the skin lesion contributed to the challenge in this diagnosis.

## Introduction

Juvenile idiopathic inflammatory myopathies (IIM) comprise a group of rare systemic diseases with a global incidence of two to four cases per million children per year [1]. These conditions are characterized by chronic inflammation of skeletal muscles, leading to progressive proximal, symmetric weakness in appendicular muscles. IIM is categorized based on clinicopathological findings into dermatomyositis (DM), overlap myositis (OM), immune-mediated necrotizing myopathy (IMNM), inclusion-body myositis (IBM), and polymyositis (PM) [2]. DM accounts for more than 80% of IIM cases and exhibits cutaneous features such as Gottron papules, heliotrope rash, or the shawl sign in addition to muscle weakness [2,3,4].

The etiopathology of IIM remains unclear, with suggestions that it may result from immune-mediated processes triggered by exposure to environmental factors such as viral, parasitic, bacterial infections, ultraviolet light, vitamin D deficiency, or drugs in genetically susceptible individuals [2,5].

Diagnosis of IIM is supported by elevated muscle enzymes, a myopathic pattern in electromyogram, and inflammation and edema findings in magnetic resonance imaging (MRI) [5]. Several autoantibodies may be present in IIM, including antinuclear, myositis-specific, and myositis antibodies, some associated with specific phenotypes and different prognostics [2,6,7]. Muscular biopsy can be performed to confirm the diagnosis when clinical and laboratory features are inconclusive [5]. The criteria for diagnosing IIM were initially proposed by Bohan and Peter in 1975, with subsequent modifications, though no official classification system exists [2,8]. The most recent validated classification, the 2017 EULAR/ACR criteria, assigns a score corresponding to the probability of having IIM [9].

IIM's management is not standardized and typically involves supportive treatment such as physical therapy, along with immunosuppression using glucocorticoids and methotrexate as the first-line therapy [2,4,10].

## Case presentation

A previously healthy seven-year-old boy presented to the Pediatric Emergency Department with a three-week progression of weakness and lower limb myalgia, limiting daily activities such as dressing up and playing. At the onset of symptoms, rhinorrhea and low-grade fever were present for two days. No other concomitant symptoms were present. There was no family history of consanguinity or neurological or rheumatic diseases.

On clinical examination, the patient was well-appearing, although seated on a wheelchair and unable to walk. He presented a gait limp with generalized muscle tenderness. A neurological exam revealed proximal muscle weakness (grade 4 according to the Medical Research Council Grading System) [11]. The remaining clinical examination was unremarkable: no facial asymmetries, unaltered tactile sensibility, tendon reflexes were all present and symmetric, muscle bulk was normal, and there were no skin rashes, including heliotrope rash or Gottron papules. Raynaud phenomenon was absent, and there was no joint involvement.

Blood tests showed an elevation of muscle enzyme levels (Table 1): aspartate aminotransferase (AST) 477 U/L, alanine aminotransferase (ALT) 268 U/L, creatinine kinase (CK) 10307 U/L, lactate dehydrogenase (LDH) 1015 U/L and myoglobin 1155 ng/mL; with negative C-reactive protein (CRP) and slight elevation of erythrocyte sedimentation rate (ESR) (25 mm/h).

**Table 1:** Laboratory investigation during the first hospitalization AST - aspartate aminotransferase; ALT - alanine aminotransferase; CK - creatinine kinase; LDH - lactate dehydrogenase; CRP - C-reactive protein; ESR - erythrocyte sedimentation rate; N/A - not assessed

Parameter	Admission	Day 34
Hemoglobin	11.6 g/dL	10.5 g/dL
Leukocytes	6180 cells/L	7050 cells/L
Neutrophils	3370 cells/L	3340 cells/L
Platelets	454,000 cells/L	444,000 cells/L
AST	477 UI/L	26 U/L
ALT	268 UI/L	30 UI/L
CK	10307 UI/L	84 UI/L
LDH	1015 U/L	285 UI/L
Myoglobin	1155 ng/ml	18.1 ng/ml
CRP	<0.05 mg/dL	N/A
ESR	25 mm/h	
Urea	21 mg/dL	
Creatinine	0.39 mg/dL	
Sodium	139 mmol/L	
Potassium	4.1 mmol/L	
Chloride	102 mmol/L	
Calcium	9.2 mg/dL	
Inorganic phosphorus	4.2 mg/dL	
Magnesium	2.0 mg/dL	
Urine analysis	Normal	Normal

The patient was hospitalized, considering the diagnosis of myositis of unknown origin, for support treatment and further investigation. During the first three days, there was a clinical deterioration, with severe bilateral muscle weakness, mostly proximal but also distal of the upper and lower limbs, without bulbar or respiratory dysfunction. On the third day of hospitalization, the boy presented a muscle weakness grade of 3/5 in the Medical Research Council Grading System, a Childhood Myositis Assessment Scale (CMAS) of 16/53 [11,12]; a positive Gowers' sign, and a myopathic gait. The remaining clinical exam was unremarkable.

Infectious, autoimmune, and metabolic investigations were performed (Table 2), with no significant changes besides a positive although low titer (1:80) of antinuclear antibody (ANA).

**Table 2:** Infectious, autoimmune, and metabolic investigation

Additional laboratory investigation		
Infectious	Respiratory viral antigen panel (Influenza A, B, and adenovirus)	Negative
	Serologies for: Epstein-Barr; cytomegalovirus; varicella zoster; parvovirus B-19; *Mycoplasma pneumoniae*, *Toxoplasma gondii*; human immunodeficiency virus (HIV); hepatits A, B, and C	Negative
	Interferon-gamma release assay (IGRA)	Negative
Autoimmune	Antinuclear antibody (ANA)	1:80
	C3, C4, CH50	Negative
	Anti-Jo, ENA (anti-SSA, SSB, RNP, Slc7o, CENP-B)	Negative
Metabolic	TSH and FT4	Normal
	Plasma and urine amino acids, urine organic acids, and plasma acylcarnitine	Normal

**Figure 1:**
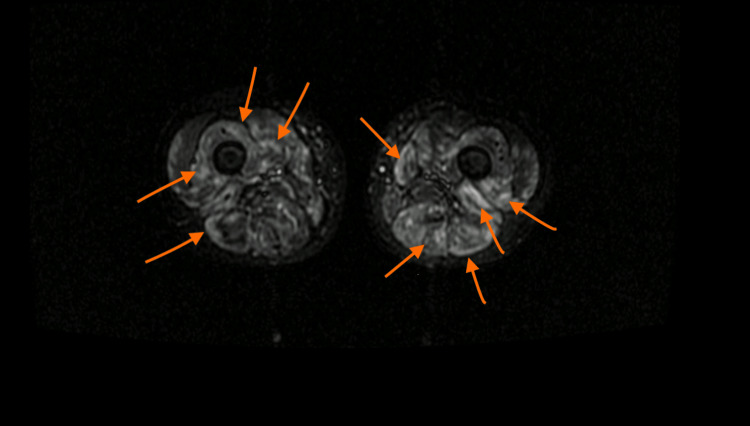
Axial STIR MRI image through patient thighs revealing heterogeneous hyperintense signal (orange arrows) affecting all the compartments STIR - short TI inversion recovery

**Figure 2:**
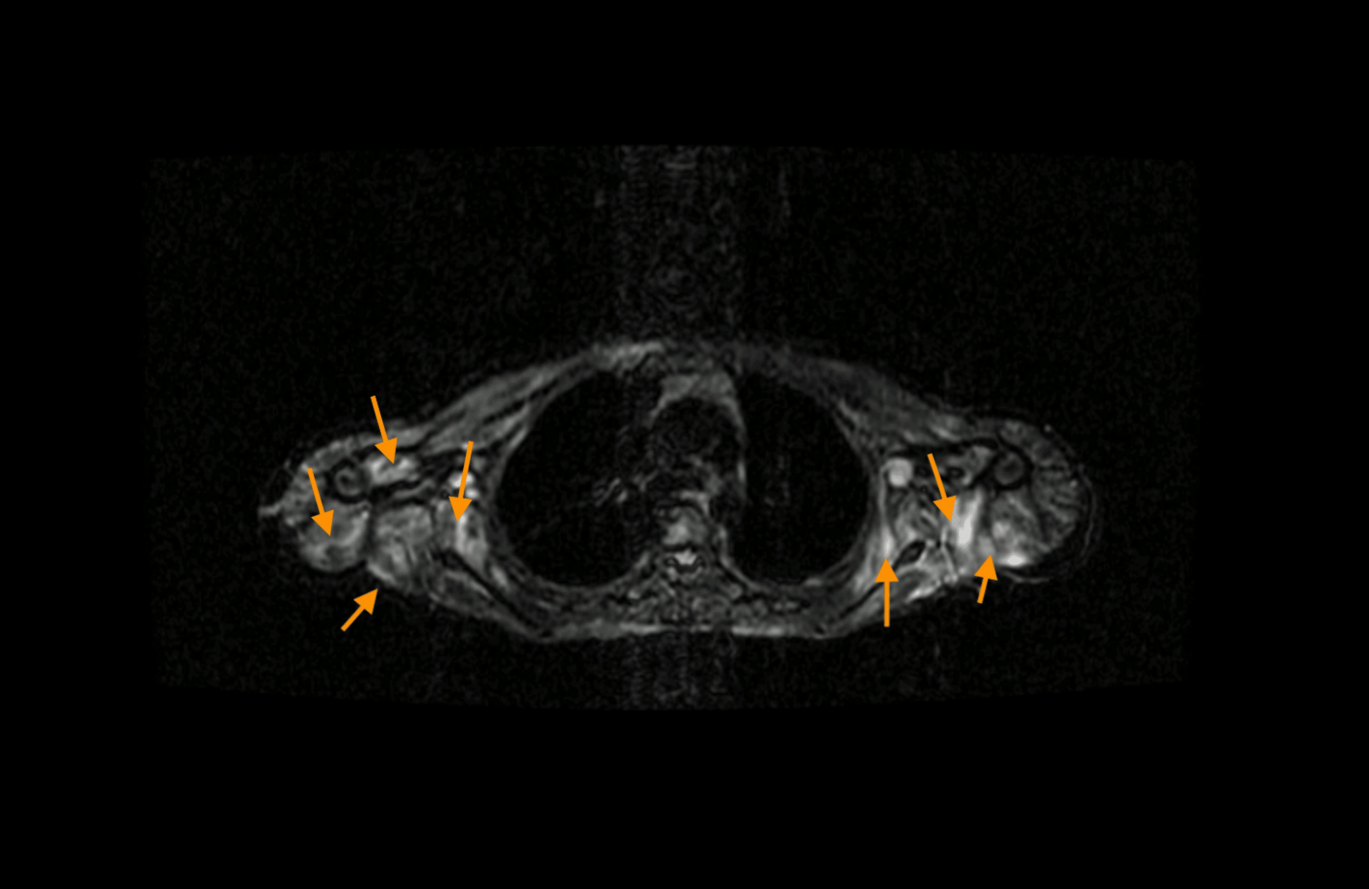
Axial STIR MRI chest image with generalized heterogeneous hyperintense signal on shoulder girdle (orange arrows) STIR - short TI inversion recovery

An electromyogram showed a myopathic pattern, and the MRI revealed generalized muscle inflammation with axial and peripheral involvement of limbs and girdles (Figures 1 and 2). The muscular biopsy was compatible with the polymyositis pattern, displaying T-cell endomysial infiltration and adjacent necrosis.

There was no involvement of other organs and systems. Echocardiogram, electrocardiogram, pulmonary function test, and abdominal ultrasound screening were all normal.

As soon as IIM was considered the most probable diagnosis (third day of hospitalization), the patient started intravenous methylprednisolone (30 mg/kg/day) for three days, followed by prednisolone (2 mg/kg/day) tapering regimen and intravenous immunoglobulin (IVIG) 2 g/kg. To prevent possible high-dose corticosteroids adverse effects, adjuvant therapy with methotrexate 15 mg/m^2^/weekly, esomeprazole 20 mg/day, folic acid 5 mg twice a week, and vitamin D3 (1.334 UI), was also used. The patient underwent a motor rehabilitation training program and after the first days of treatment, a strength improvement was evident. Following a 34-day hospitalization period, the child was discharged with increased muscle strength (CMAS 32/53) and a significant muscle enzyme reduction (Table 1).

**Figure 3:**
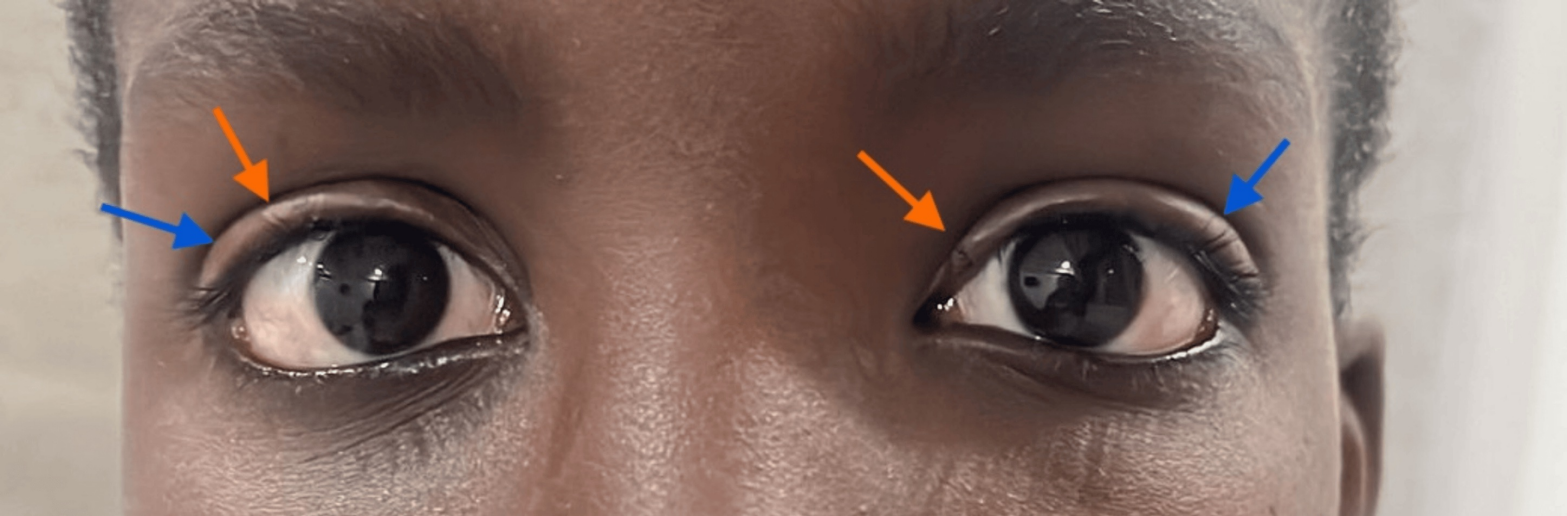
Bilateral eyelid edema (blue arrows) and epicanthi microulcers (orange arrows)

He maintained rheumatological follow-up, under prednisolone, methotrexate, and subcutaneous IG twice a week. After four months, substantial recovery was documented, with no discernible limitations in muscle strength for daily activities and normalization of muscle enzyme levels. The patient underwent a tapering regimen with prednisolone for two years and seven months. A relapse occurred three years post-remission (eight months after stopping prednisolone) characterized by the onset of left hemiface edema, bilateral epicanthi microulcers (Figure 3), muscle weakness (CMAS 49/52) and muscle enzyme elevation (Table 3) - AST 905 U/L, ALT 363 U/L, CK 29 061 U/L, myoglobin 1253 ng/mL, with a negative viral myositis panel.

**Table 3:** Laboratory investigation during the relapse (second hospitalization) AST - Aspartate aminotransferase, ALT - Alanine aminotransferase, CK - Creatinine kinase, CRP - C-reactive protein, ESR - Erythrocyte sedimentation rate, N/A - Not assessed.

Parameter	Admission	Day 5 of hospitalization (discharge)	2 months after hospitalization (60th day)
Hemoglobin	13.8 g/dL	10.5 g/dL	13.1 g/dL
Leukocytes	5280 cells/L	8730 cells/L	8550 cells/L
Neutrophils	3180 cells/L	4180 cells/L	1110 cells/L
Platelets	368 000 cells/L	370 000 cells/L	355 000 cells/L
AST	905 UI/L	153 UI/L	28 UI/L
ALT	363 UI/L	169 UI/L	28 UI/L
CK	2906 UI/L	816 UI/L	142 UI/L
CRP	<0.05 mg/dL	N/A	N/A
ESR	21 mm/h	N/A	N(A
Urine analysis	Normal	N/A	N/A

The patient was hospitalized, treated with corticosteroid (methylprednisolone 30 mg/kg followed by 1mg/kg prednisolone) and IVIG 2 g/kg, and discharged after four days, with favorable clinical and laboratory evolution, with normalization of muscle enzymes two months after (Table 2).

Evidence of an isolated malar erythematous papule five months later in a follow-up appointment. The patient restarted a slow taper of maintenance steroids, with posterior suspension after one year of treatment. Until this date, no following crises were observed.

## Discussion

Dermatomyositis is rare in the pediatric population, and its diagnosis is challenging, demanding the exclusion of several diseases that share the same clinical features, including other IIM, infectious, metabolic, endocrine, toxic, systemic diseases related and inherited myopathies [2,4,13,14]

This child revealed clinical features compatible with myopathy (myalgia, muscle weakness, muscle enzyme elevation without neuropathy features), confirmed with myopathic pattern on the electromyogram. The absence of a myopathy family history made inherited myopathies less likely. After the exclusion of infectious causes (normal white blood count, negative C-reactive protein, negative viral panel) and other secondary causes (toxic substances, medication, endocrinopathy, or systemic disease), an idiopathic inflammatory myopathy was considered the most probable diagnosis. Diffuse muscle inflammation in MRI, subacute proximal weakness, and positive ANA were consistent with IIM. Considering IIM's clinical and analytical shared features, a muscular biopsy was performed. Polymyositis biopsy findings, coupled with the absence of DM's typical lesions in the inaugural crisis, contributed for the juvenile polymyositis (JPM) initial diagnosis [2,10,14].

Three years after remission, bilateral eyelid microulcers, an isolated malar papule, and myositis emerged. Therefore, juvenile dermatomyositis (JDM) was considered the most probable diagnosis. JDM presentation may be heterogeneous. In this case specifically, the patient presented isolated muscle weakness at the inaugural episode. Dermatomyositis' pathognomonic skin rashes (Gottron papules and heliotrope rash) have been identified as one of the most crucial early diagnostic criteria. However, according to the literature, these characteristic rashes are not recognized in 12% of children under 18 years old at initial presentation and symptom onset [15]. This child revealed an atypical skin presentation (eyelid ulcers) years after the inaugural crisis. A myriad of non-hallmark skin manifestations with varying specificity degrees have emerged as featuring clues to diagnosing DM, promoting to further expansion of the known DM phenotype [16]. Skin ulceration is frequently a sign of more extensive vasculopathy and predicts a more serious disease [13,16]. It may develop over flexor surfaces, the trunk, or a medial canthus of the eyelid. 

Specific and associated myositis autoantibodies negative values, besides positive antinuclear autoantibodies in this case, are consistent with literature information since they have only been identified in approximately 40% of pediatric patients with IIM [6,7,16]. This case also highlights the need for further studies in myositis autoantibodies since currently unknown antibodies with higher sensitivity and specificity for JDM may be involved. With autoimmunity screening and MRI's increasing use as an IIM diagnosis tool in pediatric age, muscle biopsy is being less employed [2,3]. Identifying these markers, combined with expanding the criteria for diagnosing probable JDM to include children without rash, could improve early diagnosis and outcomes for JDM in the future [15]. 

The prognosis of juvenile inflammatory myopathies is generally good. However, morbimortality associated with IIM can be significant, especially in the presence of systemic involvement (fever, arthralgias, Raynaud's phenomenon, arrhythmia, dysphagia, interstitial lung disease) [4,10,14]. In this case, the absence of multisystem involvement allied with a multidisciplinary approach, prompt diagnosis, early treatment with corticosteroids, and an intensive motor rehabilitation program were all important contributors to this patient's favorable outcome [5,16]. Juvenile dermatomyositis' prognosis is variable, with an estimated overall mortality rate of 2-3%. Around 50% of patients with IIM have chronic disease, and 25% have a polyphasic course with complete remission and recurrence periods [5,13].

## Conclusions

Juvenile dermatomyositis (JDM) is rare in children, and clinical and scientific evidence of this disease in this age group is sparse. This condition should be considered in a patient with subacute onset of bilateral proximal muscle weakness, skin lesions, muscle cytolysis, and myositis findings in electromyogram and MRI.

This report underlines the challenging diagnosis in this patient due to late skin manifestation onset and its broad differential diagnosis, including other inflammatory idiopathic, infectious, and inherited myopathies. A high index of suspicion for this disease with prompt diagnosis and treatment is crucial to a favorable clinical course.

However, the significant gaps in knowledge regarding IIM in pediatric age, concerning epidemiological features, pathogenesis, treatment, and functional outcomes, constituted a limitation in this case specifically. Despite access to specialized and multidisciplinary team care, the time course from initial evaluation to MRI and muscle biopsy was prolonged, reflecting the difficulty in establishing an IIM diagnosis. Another limitation in this case was the presence of negative myositis-associated and specific antibodies, which could have helped to predict clinical features, treatment response, and prognosis. 
